# Cross-cultural French adaptation and validation of the Impact On Family Scale (IOFS)

**DOI:** 10.1186/1477-7525-11-67

**Published:** 2013-04-23

**Authors:** Raphaël Boudas, Jérémie Jégu, Bruno Grollemund, Elvire Quentel, Anne Danion-Grilliat, Michel Velten

**Affiliations:** 1 Department of Epidemiology and Public Health, EA 3430, Faculty of Medicine, University of Strasbourg, 4 rue Kirschleger, Strasbourg 67000, France; 2 Department of Public Health, University Hospital of Strasbourg, 1 Place de l’Hôpital, Strasbourg 67000, France; 3 Orthodontic Department, Faculty of dentistry, University of Strasbourg, 1 Place de l’Hôpital, Strasbourg, France; 4 Laboratoire éthique et pratiques médicales, IRIST EA 3424, Université de Strasbourg, Rue de l’Université 7, Strasbourg, France; 5 Service Psychothérapique pour Enfants et Adolescents, Hôpitaux Civils, Hôpitaux Universitaires de Strasbourg, Place de l’Hôpital 1, Strasbourg 67000, France; 6 Department of Epidemiology and Biostatistics, Paul Strauss Comprehensive Cancer Center, 3 rue de la porte de l’hôpital, Strasbourg 67000, France

**Keywords:** Quality of life, Family, Child, Cleft lip, Cleft palate, Validation studies

## Abstract

**Background:**

The IOFS (Impact On Family Scale) questionnaire is a useful instrument to assess the impact of chronic childhood conditions on general family quality of life. As this instrument was not validated in French, we proposed to translate, adapt and validate the IOFS questionnaire for clinical and research use in French-speaking populations.

**Findings:**

The sample studied comprised French-speaking parents with a child presenting a cleft lip or cleft lip and palate, aged 6 to 12 years and treated in the University Hospital of Strasbourg, France. The 15-item version of the IOFS was translated into French and then sent to the parents by post. The structure of the measure was studied using Exploratory Factor Analysis (EFA), internal consistency was assessed using Cronbach’s alpha coefficient and test-retest reliability was studied by calculating the Intraclass Correlation Coefficient (ICC).

A total of 209 parents answered the questionnaire. Its acceptability was good, with 67.9% of mothers and 59.9% of fathers answering the questionnaire. EFA identified one main factor that explained 77% of the variance. Internal consistency was good, with a Cronbach’s alpha of 0.93. Finally, the ICC values were 0.77 (95% confidence interval 0.66–0.85) and 0.87 (95% confidence interval 0.80–0.92) for inter- and intra-observer reliability respectively.

**Conclusions:**

The French version of the IOFS questionnaire exhibited very good psychometric properties. For practitioners, this instrument will facilitate the assessment of the impact of chronic childhood conditions on quality of life among French-speaking families.

## Background

Quality of life measurement has become a major topic in pediatric medicine alongside the advances in medical care of children with chronic conditions [1]. In particular, chronic conditions have been reported to affect family functioning and family well-being [2]. The IOFS (Impact On Family Scale) questionnaire is an instrument measuring health and quality of life which enables quantification of the impact on the family of chronic childhood conditions. This instrument, initially developed by Stein et al. with 33 items and 4 dimensions [3] was progressively reduced to a 15-item questionnaire with one main dimension representing general negative impact on the social and familial systems [4,5]. To date, validations of cross-cultural adaptations of the IOFS have been performed in different languages: Spanish, Italian, German, Portuguese and Turkish [6-10]. As this instrument was still not validated in French, we proposed to translate, adapt and validate the IOFS questionnaire for clinical and research use in French-speaking populations.

## Methods

The guidelines proposed by Beaton et al. were used for the cross-cultural adaptation of the 15-item IOFS questionnaire [11]. Briefly, the IOFS was translated into French by two independent native French translators. A synthesis of the two questionnaires obtained was performed by an expert committee. Then the resulting French questionnaire was back-translated into English by two independent English-native translators and the two questionnaires obtained were reviewed by the expert committee. Finally, the French-translated questionnaire obtained was pre-tested on 30 people to ensure that the questionnaire was perfectly understandable and clear.

The population included during the validation process consisted of French-speaking parents with a child presenting a cleft lip, or a cleft lip and cleft palate, treated in the French University Hospital of Strasbourg. The children included were to be aged from 6 to 12 years and were not to have undergone surgery in the past 12 months.

The French-translated IOFS questionnaires were sent to the parents by post. These questionnaires were accompanied by a short questionnaire about the parents’ socio-professional characteristics. Parents were asked to return completed questionnaires to the investigation center by post. Finally, a second IOFS questionnaire was sent 15 days after the first one for the purpose of inter-observer reliability assessment. In addition, clinical data about the children (gender, age and type of cleft) were collected using the University Hospital of Strasbourg patient database.

Acceptability was assessed on the basis of the proportion of parents answering the first IOFS questionnaire. The total impact score was calculated by summing the results of all items, giving a total score ranging from 15 (no impact) to 60 (maximum impact). It should be noted that at this step of the analysis, we had to reverse the item values attributed (1 becoming 4, 2 becoming 3 and so on…) to obtain higher IOFS score values for greater impact. Median and inter-quartile ranges were used to describe IOFS total score distribution. Then the structure of measure was studied using an Exploratory Factor Analysis (EFA), a variable reduction technique which identifies the number of latent constructs and the underlying factor structure of a set of variables [12]. The internal consistency of the measure was assessed by calculating Cronbach’s alpha coefficient. The children were considered as the statistical unit (n = 113) for EFA and internal consistency assessment. If both parents answered to the questionnaire, we restricted the analyses to the answers provided by the mother, as the answers from parent couples could not be considered independent. Finally, inter-observer reliability (between parents of the same child) and intra-observer reliability (between the two questionnaires completed within 15 days of each other) were assessed by calculating the Intraclass Correlation Coefficient (ICC). All analyses were performed using SAS version 9.2 (SAS Institute, Cary, North Carolina).

## Results

A total of 327 questionnaires were sent by post to the parents of the children included. Among the parents contacted, 209 (63.9%) completed and returned the IOFS. Acceptability was slightly better among mothers compared to fathers, with response rates of 67.9% and 59.9% respectively. A minority of parents were divorced or single (28.4% of the mothers and 22.6% of the fathers), the educational level was higher among mothers compared to fathers (49.5% had a higher education diploma versus 32.6%) and a majority of parents had an individual monthly income between 1500 and 3000 € (40.4% among mothers and 48.9% among fathers). Concerning the 113 children included, there was a majority of boys (65.5%), the mean age was 8.5 years (SD 1.8) and most of them presented a unilateral cleft lip and cleft palate (52.2%). The median score on the IOFS was 17 with an inter-quartile range of 15–24.

EFA, which was not restricted at the outset for the number of factors, identified one main factor which explained 77% of the variance, followed by a second one which explained only 10% of the variance. The results of factor loadings obtained by restricting EFA to one factor are shown in Table 1. The items presenting the highest loadings were: “family gives up things” (IMPT), “see family and friends less” (IMPK), “fatigue is a problem” (IMPU) and “hard to find reliable person to care for child” (IMPI).

**Table 1:** Factor loadings of fifteen Impact on Family Scale items; one-factor principal component solution (n = 113)

**Item label**	**Text (abbreviated)**	**Item loading**
IMPU	Fatigue is a problem	0.81
IMPK	See family and friends less	0.83
IMPJ	Need to change plans at last minute	0.76
IMPH	Little desire to go out	0.71
IMPR	No time for other family members	0.75
IMPV	Live from day to day	0.67
IMPI	Hard to find reliable person to care for child	0.81
IMPT	Family gives up things	0.88
IMPW	Nobody understands the burden	0.73
IMPF	Can’t travel out of city	0.70
IMPAA	Live on roller coaster	0.74
IMPG	People treat us special	0.73
IMPX	Travel to hospital is a strain	0.58
IMPO	Think about not having more children	0.49
IMPM	Wonder whether to treat child “specially”	0.65

The assessment of internal consistency yielded a Cronbach’s alpha of 0.93.

Finally, ICC values were 0.77 (95% confidence interval 0.66–0.85) and 0.87 (95% confidence interval 0.80–0.92) for inter- and intra-observer reliability respectively.

## Discussion

The French version of the IOFS questionnaire showed very good psychometric properties. During the validation process, EFA identified one main factor explaining most of the variability, which is consistent with the results previously published by Stein et al. and Williams et al. for the 15-item version of the IOFS [4,5]. The results of factor loadings obtained in our study differed slightly from those reported in these articles. This could be explained by the range of physical disorders among children included in the Pediatric Ambulatory Care Treatment Study (PACTS), the Sharing The Experience of Parenting study (STEP), the Family Advocacy and Coordination Effort study (FACE) and the Intervention for Siblings study (ISEE), compared to the homogenous population of children presenting a cleft lip, or a cleft lip and a cleft palate considered in our study. However, it should be noted that the items “See family and friends less” (IMPK) and “Fatigue is a problem” (IMPU) nevertheless presented among the highest loadings in our study, consistent with the results obtained with the PACTS, STEP, FACE and ISEE datasets. The internal consistency of the French-adapted IOFS was very good (Cronbach’s alpha was 0.93), which agrees with the estimates of 0.89, 0.88 and 0.83 in PACTS, STEP and FACE validation studies respectively [4]. Finally, test-retest reliability was good, with an ICC of 0.87, which can be compared to the 0.94 ICC estimate for the general impact dimension in the Turkish version of the IOFS [10].

In conclusion, the French IOFS questionnaire is a reliable instrument to assess the impact of chronic childhood conditions on quality of life among French-speaking families. This instrument will be of immediate use in an ongoing prospective, multidisciplinary and multi-centre French study aiming to explore the perceptions and feelings of parents in the year following the birth of a child with cleft lip and palate, and to analyse parent–child relationships [13].

## Abbreviations

IOFS: Impact on family scale; ICC: Intraclass correlation coefficient; EFA: Exploratory factor analysis; PACTS: Pediatric ambulatory care treatment study; STEP: Sharing the experience of parenting; FACE: Family advocacy and coordination effort; ISEE: Intervention for siblings.

## Competing interests

The authors declare that they have no competing interests.

## Authors’ contributions

RB made substantial contributions to the conception and the design of the cross-cultural adaptation and validation of the questionnaire and wrote the first draft of the article. JJ performed the statistical analyses, data interpretation and revised the manuscript. BG coordinated the study and the data collection and revised the manuscript. EQ participated in all stages of this study, and was particularly involved throughout the data collection process. ADG revised the manuscript. MV participated in the study management, statistical analysis and revised the manuscript. All authors have read and approved the final manuscript.
